# Fibrin-Targeting Immunotherapy for Dementia

**DOI:** 10.14283/jpad.2023.105

**Published:** 2023

**Authors:** A.B. Kantor, K. Akassoglou, J.B. Stavenhagen

**Affiliations:** 1. Therini Bio, Inc, Sacramento, CA, USA;; 2. Gladstone Institute of Neurological Disease, San Francisco, CA, USA;; 3. Department of Neurology, Weill Institute of Neurosciences, University of California, San Francisco, San Francisco, CA, USA

**Keywords:** Fibrinogen, fibrin, neuroinflammation, neurodegeneration, microglia, Alzheimer’s disease

## Abstract

Blood-brain barrier (BBB) disruption is an early event in the development of Alzheimer’s disease. It precedes extracellular deposition of amyloid-β in senile plaques and blood vessel walls, the intracellular accumulation of neurofibrillary tangles containing phosphorylated tau protein, microglial activation, and neuronal cell death. BBB disruption allows the coagulation protein fibrinogen to leak from the blood into the brain, where it is converted by thrombin cleavage into fibrin and deposits in the parenchyma and CNS vessels. Fibrinogen cleavage by thrombin exposes a cryptic epitope termed P2 which can bind CD11b and CD11c on microglia, macrophages and dendritic cells and trigger an inflammatory response toxic to neurons. Indeed, genetic and pharmacological evidence demonstrates a causal role for fibrin in innate immune cell activation and the development of neurodegenerative diseases. The P2 inflammatory epitope is spatially and compositionally distinct from the coagulation epitope on fibrin. Mouse monoclonal antibody 5B8, which targets the P2 epitope without interfering with the clotting process, has been shown to reduce neurodegeneration and neuroinflammation in animal models of Alzheimer’s disease and multiple sclerosis. The selectivity and efficacy of this anti-human fibrin-P2 antibody in animal models supports the development of a monoclonal antibody drug targeting fibrin P2 for the treatment of neurodegenerative diseases. THN391 is a humanized, affinity-matured antibody which has a 100-fold greater affinity for fibrin P2 and improved development properties compared to the parental 5B8 antibody. It is currently in a Phase 1 clinical trial.

## Introduction

**A**lzheimer ‘s disease (AD) is a progressive neurodegenerative pathology leading to dementia and death. Two pathological hallmarks characterize AD: extracellular amyloid beta (Aβ) plaques, and intraneuronal neurofibrillary tangle (NFT) deposits, which contain altered hyperphosphorylated tau protein. Aβ deposition is common to cerebral amyloid angiopathy (CAA), where it occurs in the brain vasculature, and AD, where it occurs in the brain parenchyma (1). The two pathologies often coexist in the same patients (2), and CAA is a major factor in AD severity (3). Anti-Aβ monoclonal antibodies (mABs) are currently the leading disease-modifying therapies for AD. Passive immunotherapy with aducanumab, lecanemab, and donanemab all lower Aβ burden, but are only partially effective in slowing cognitive decline (4–6). The recent approval of aducanumab and lecanemab, and pending approval of donanemab, has generated promise for patients and families suffering from AD. However, lowering Aβ burden does not fully protect from cognitive decline, suggesting that additional pathways need to be targeted. Furthermore, some of the known risks of anti-Aβ treatments may limit use of this class of drugs. Patients have a significant risk of amyloid-related imaging abnormality (ARIA). The incidence of ARIA-E (cerebral edema) has varied from 0.9 to 42%, and the incidence of ARIA-H (cerebral microhemorrhages) has varied from 0.5 to 31%. The risk of ARIA and amyloid clearance both increase in a dose-dependent manner, and APOE4 carriers are more susceptible to ARIA risk. Currently, the only recommendation for treatment of symptomatic ARIA is to reduce the dose or discontinue treatment (recently reviewed (5, 7–9)).

The amyloid cascade alone does not completely explain AD pathogenesis. An array of mechanisms, including neuroinflammation, vascular defects, oxidative stress, mitochondrial dysfunction, cholesterol and fatty acid metabolism, glucose pathway impairments, dysfunction of acetylcholine-containing neurons, and autophagy failure may also contribute to AD (10–12). This multifactorial nature of the disease suggests the need for alternative and combination therapies. As of January 2023, there were 147 unique AD drugs in clinical trials, with the most common targets being transmitter receptors, amyloid, synaptic function, and inflammation (13). Targets related to inflammation comprise one of the largest categories, representing 20% of the drugs in the pipeline, with 6, 17 and 3 drugs in Phase I, II and III, respectively. Most anti-inflammatory agents have targets within the inflammatory cascade (13, 14). Uniquely, Therini Bio recently launched the clinical development of THN391, a first-in-class fibrin-targeting therapeutic candidate for AD (15). In this review, we focus on the rationale for this disease-modifying therapy, including breakdown of the blood-brain barrier (BBB) and the contribution of fibrin(ogen) to neurodegeneration via neurotoxic polarization of microglia and macrophages.

## BBB dysfunction in Alzheimer’s disease: Neuropathology and biomarker studies

The BBB is a continuous, tightly sealed monolayer of endothelial cells sheathed by vascular pericytes in capillaries and smooth muscle cells in the arterioles and arteries, along with perivascular astrocyte end-feet. In healthy brains, this neurovascular unit (NVU) prevents the entry of most blood-derived components and pathogens into the brain unless specific carriers enable their transport. BBB breakdown is associated with normal aging and inflammatory and immune responses, which can initiate multiple neurodegenerative pathways. In normal aging, vascular pathology is an independent predictor of cognitive decline that also acts synergistically with Aβ burden (16, 17). Vascular dysfunction may be an early biomarker for assessing risk of prospective cognitive decline in preclinical AD (17). Indeed, early BBB breakdown and dysfunction occur in AD and dementia patients before neurodegeneration and brain atrophy (18–20).

There are three classes of evidence connecting BBB breakdown to AD: 1) post-mortem brain histology and bioanalytical assays, 2) non-invasive neuroimaging, and 3) cerebral spinal fluid (CSF) component analysis. Post-mortem analysis of AD patients often shows vascular pathology, including presence of large infarcts, lacunes and multiple microinfarcts, hemorrhages, atherosclerosis, arteriosclerosis, and CAA (21–23). Multiple histochemical studies of brain tissue from AD patients have found capillary leakage of blood-derived proteins in the prefrontal and entorhinal cortex and hippocampus, including accumulation of fibrinogen, thrombin, albumin, IgG, and iron-containing proteins such as hemosiderin (24–33). AD-associated Aβ deposits are often co-localized with these blood derived proteins (27–29, 32–34).

Positron emission tomography (PET) and magnetic resonance imaging (MRI) have been used extensively to evaluate BBB integrity and function in AD and other neurodegenerative disorders. For example, these approaches can be used to track the uptake of intravenously injected tracers as they leak from the bloodstream into the brain through the BBB. In addition, PET detects lower expression of glucose transporter 1 (GLUT1) and P-glycoprotein (P-gp) in the BBB of mild cognitive impairment (MCI) and AD patients. Different radiolabeled tracers, such as (18)F-FDG and [11C]-verapamil, detect changes in both energy metabolism and Aβ clearance (35–37). FDG-PET shows that GLUT1 levels are substantially diminished in brain micro-vessels in AD (19, 38), Moreover, imaging has revealed that patients with MCI have reduced glucose uptake in multiple brain areas prior to any apparent neurodegenerative changes, brain atrophy or conversion to AD (39, 40).

Dynamic contrast-enhanced (DCE) MRI with gadolinium contrast agents has a higher spatial resolution than PET and enables the BBB permeability constant (Ktrans) to be quantified (41). DCI-MRI shows increased BBB permeability in the hippocampus of patients with MCI compared with normal controls (42, 43). Moreover, BBB permeability of the medial temporal lobe is an independent early imaging biomarker of cognitive impairment unrelated to Aβ or tau pathology (43) and global BBB leakage found in patients with early AD is associated with cognitive decline (44). There is also a global decrease in cerebral blood flow in the gray matter of patients with early AD, which was correlated with increased BBB leakage (45). APOE4, the strongest risk factor gene for AD, is also related to increased BBB permeability in both patients with MCI and cognitively normal controls, supporting involvement of BBB dysfunction early in the development of AD (46–48).

The amount of blood proteins in the CSF can indicate BBB breakdown, but their quantitative measurement should include paired plasma. Indeed, a reference for the evaluation of blood-derived proteins in the CSF is QAlb, the CSF/serum concentration quotient of albumin, as it reflects individual biological variations in diffusion pathways at the blood-brain and brain-CSF interfaces, the individual CSF flow rate and the lengths of flow ways (49). QAlb is generally, but not always, reported to be elevated in patients with preclinical AD, MCI, and AD (42, 50–53). CSF/serum IgG index and CSF plasminogen have also been associated with BBB disruption in neurodegenerative disorders, however, the significance of these findings still needs further clarification (19, 54). Furthermore, soluble platelet-derived growth factor receptor-β (sPDGFRβ) is a marker of pericyte injury (42, 55). CSF levels of sPDGFRβ may represent a direct and sensitive marker of neurovascular unit damage. Higher levels of CSF PDGFRβ are associated with the severity of clinical symptoms and brain vascular damage when AD was diagnosed by clinical symptoms and CSF biomarkers (43, 56). Correlation of PDGFRβ levels to clinical disease severity was especially strong in APOE4 allele carriers (46, 57). However, studies linking sPDGFRβ in CSF to AD have had mixed results, potentially because they rely on different measurement approaches that have not been validated (43, 46, 58–61). CSF fibrinogen levels are reported to correlate with QAlb and sPDGFRβ (43, 46, 59, 60). QFib, the CSF/plasma fibrinogen quotient, is higher in people with migraines (59), and is likely to be a more robust marker for BBB disruption than CSF alone in dementia.

## BBB breakdown and fibrin deposition in the AD brain

**Figure 1.**
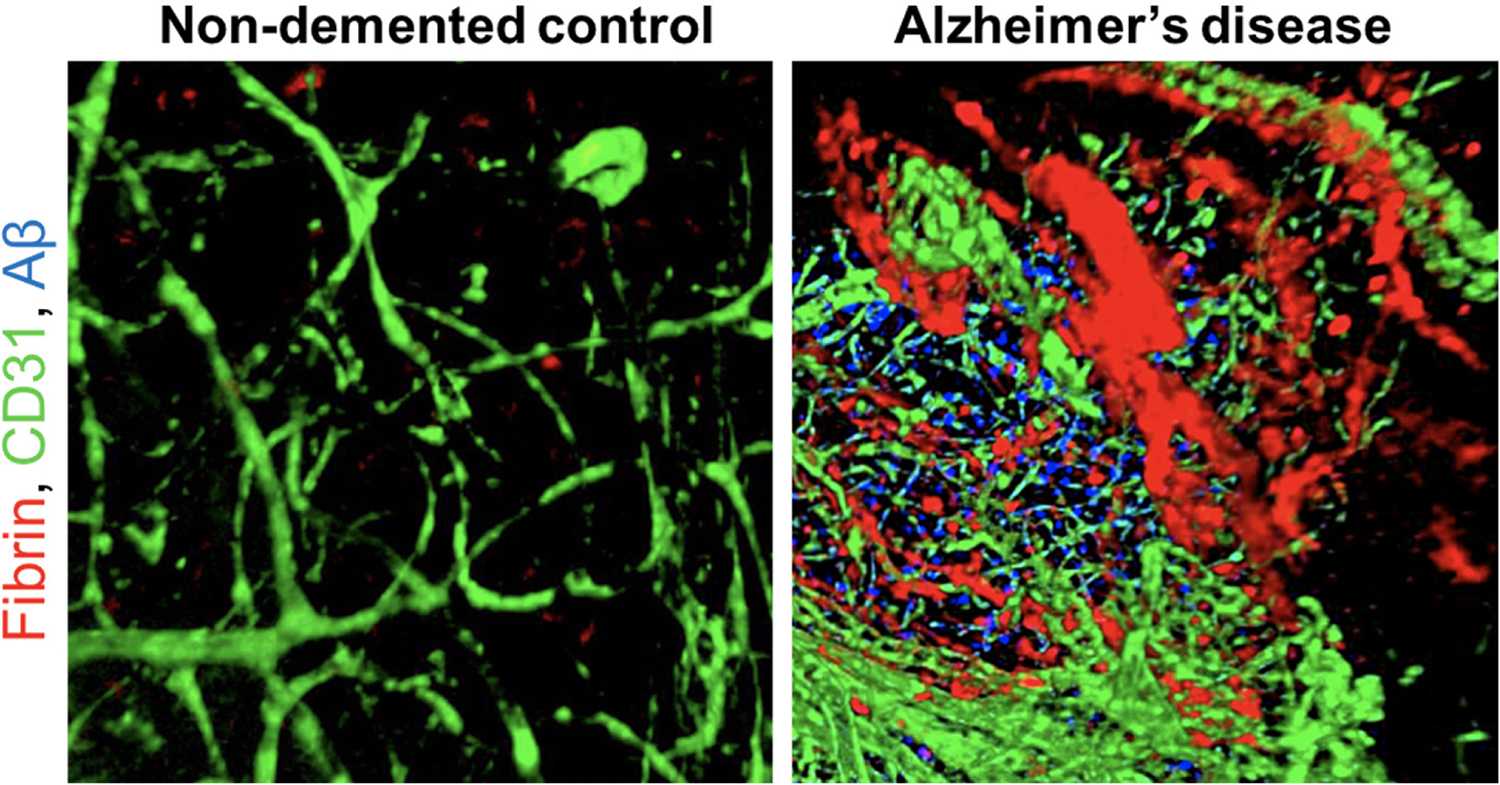
Fibrin is deposited in the brain of individuals with Alzheimer’s disease Confocal 3D volume projections of lateral temporo-occipital cortex of AD patients and age-matched non-demented control subjects acquired with confocal microscopy showing fibrin (red), CD31-positive blood vessels (green), and Aβ plaques (6E10 antibody, blue). Reprinted from Publication Neuron, 101 (6) Merlini et al. Fibrinogen Induces Microglia-Mediated Spine Elimination and Cognitive Impairment in an Alzheimer’s Disease Model 1099–1108, Copyright (2019), with permission from Elsevier.

The presence of fibrinogen and fibrin in the brain and CSF of dementia patients is a potential early marker of dementia (Table 1). In AD patients and mouse models of AD, leaked fibrinogen (coagulation factor I) is converted into fibrin by thrombin and deposited in the brain parenchyma and CNS vessels (24, 28, 62–67). In both brains from post-mortem AD patients and mouse models of AD, fibrin deposits are detected both at sites of Aβ plaques and independent of Aβ within the brain parenchyma, as well as around blood vessels (32, 33, 62, 67) (24, 32, 33, 62, 67) (Figure 1). CAA, which is frequently observed in AD patients and asymptomatic aged adults (68–70), results from Aβ deposits in and around cerebral blood vessels that are believed to mediate disruption of the brain vasculature. In vivo and in vitro experiments demonstrate that fibrinogen binding to Aβ affects fibrin clot formation and structure and inhibits fibrinolysis (71–74). Aβ binds to the fibrinogen β chain and αC region resulting in fibrin clots resistant to plasmin-mediated cleavage leading to persistent fibrin deposition (71–74). Fibrin(ogen) associates with Aβ around blood vessels in the TgCRND8 APP mouse model, where it deposits in the parenchyma and correlates with neuronal death and overall AD pathology (62). Fibrin(ogen) is present as cortical and parenchymal deposits in 5XFAD and hAPP-J20 mice that increase with age (32). The Dutch and Iowa Aβ variants are linked to hereditary CAA and have a 50-fold stronger affinity for fibrinogen that leads to perturbation of fibrin networks and a significant reduction in fibrinolysis. Deposition of Aβ /fibrin(ogen) is significantly elevated within and around the brain vasculature in these hereditary CAA patients (66).

Most of these previous studies used antibodies that bind to both fibrinogen and insoluble fibrin (IFib) or used extraction procedures that remove all soluble fibrinogen and then stain to demonstrate increased amounts of IFib in AD brains. A recent study used a highly specific anti-IFib antibody, MAb 102–10, that does not bind fibrinogen, soluble fibrin, or D-dimer (75). Anti-IFib MAb 102–10 recognizes the hydrophobic structure on the Bβ chain that appears only after fibrin formation (76). In an AD mouse model, this antibody showed IFib deposited around and inside blood vessel walls, and colocalized with Aβ in the small capillaries and arterioles. The amount of both proteins in lesions positively correlated with disease stage and severity (77).

Although fibrin(ogen) is below the limits of detection outside of blood vessels in the healthy CNS, it is abundantly deposited in a wide range of neurological diseases and traumatic injuries associated with BBB disruption (19, 65, 78). High levels of fibrinogen in plasma correlate with increased risk for dementia (28, 79, 80). Indeed, plasma fibrinogen correlates plasma Aβ40 and Aβ42 and CSF phosphorylated tau-181 (p-tau), as well as indicators of Aβ deposition in the brain, such as p-tau/Aβ42, and with neocortical amyloid burden (81, 82). Thus, and fibrinogen in CSF (83–85) and plasma (86, 87) may serve as a useful biomarker to identify early stages in AD progression and to monitor response to treatment.

## Fibrinogen and fibrin structure and function

Fibrinogen is the major plasma protein coagulation factor, with a normal concentration range of 2–4 mg/mL (5–10 mM). It circulates in the blood as a large rod-like soluble glycoprotein with a typical molecular weight of ~340-~420 kDa. It is composed of six polypeptide chains: two Aα, two Bβ and two γ chains. The final secreted, hepatocyte-derived protein consists of two disulfide linked trimers, each composed of an Aα, Bβ and γ chain (88, 89).

Activation of the coagulation cascade leads to the conversion of fibrinogen to fibrin. Thrombin cleaves fibrinopeptide A and B from fibrinogen revealing polymerization sites (knobs and holes), which enable fibrin to assemble into an interlocking network and begin clot formation. Fibrin interacts with platelets, endothelial cells and leukocytes, enabling hemostasis, thrombosis, and inflammatory responses (89). Fibrinolysis, mediated by tissue-plasminogen activator (tPA)/plasmin, induces fibrin degradation to resolve blood clots and dynamically remodel fibrin matrices in tissues (90). In neurologic diseases, fibrinolysis is impaired, by either upregulation of inhibitors of plasmin generation, such as plasminogen activator inhibitor 1 (PAI-1), or by Aβ binding to the plasmin-binding site on fibrin inhibiting its degradation (24, 90). Impaired fibrinolysis can cause excessive and persistent fibrin deposits with detrimental outcomes on chronic inflammation and inhibition of tissue repair (90, 91).

Fibrinogen conversion to fibrin exposes a cryptic sequence on the γ chain, at amino acids 377–395, known as fibrin P2. Subsequent binding of the exposed fibrin P2 sequence to the α-I -domain on CD11b/CD18 (also known as Mac-1, complement receptor 3 (CR3), and αMβ2 integrin receptor) on microglia, macrophages, neutrophils and dendritic cells triggers an inflammatory response (63, 92–96), leading to oxidative stress and secretion of cytokines that damage nerves (32, 97–99). Fibrin P2 also binds the α-I domain on CD11c. CD11c (also known as integrin αX) is a defining marker for dendritic cells. When paired with CD18, the heterodimeric receptor (CR4, αXβ2, CD11c/ CD18) binds to complement iC3b and mediates phagocytosis (100). Biophysical studies suggest that varying ratios of CD11b and CD11c contribute to cell-type specific functions (101, 102). A subset of innate immune cells that expresses CD11c appear early in life and expand in murine models of neurodegenerative disease, including AD (103–107).

**Figure 2.**
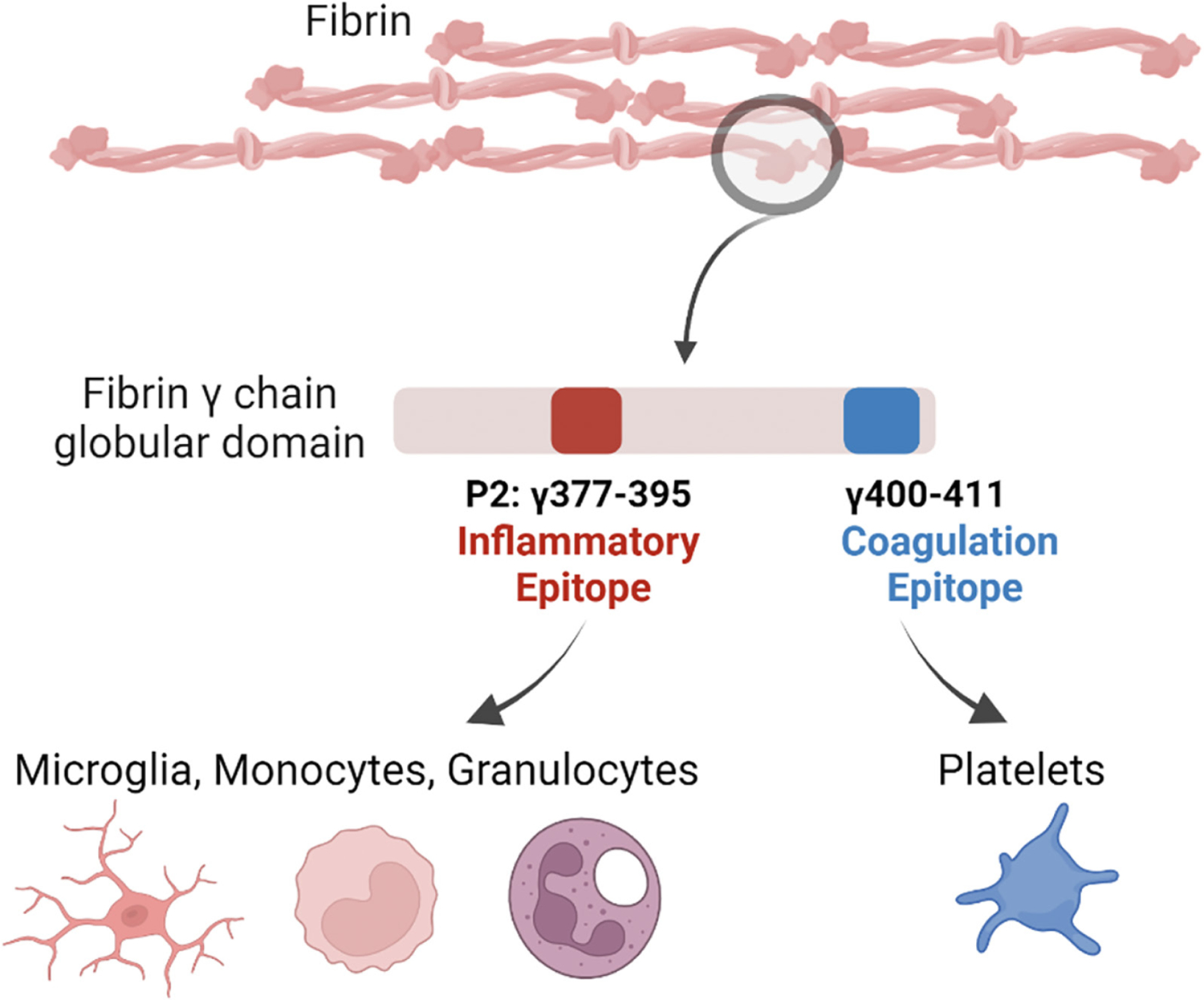
Fibrin-driven inflammation in chronic disease is distinguishable from its role in hemostasis Conversion of the blood coagulation protein fibrinogen to fibrin by thrombin exposes a cryptic sequence on the γ chain at amino acids 377–395, known as fibrin P2. This inflammatory P2 epitope is spatially and compositionally distinct from the coagulation epitope, which is located on the γ chain at amino acids 400–411. Created with BioRender.com.

The inflammatory P2 epitope is spatially and compositionally distinct from the coagulation epitope, γ400–411 ((108), Figure 2), which is a binding site for the platelet integrin receptor αIIbβ3 required for platelet aggregation and hemostasis. Thus, 390–396A mice, which harbor alanine substitutions for the last 7 amino acids of P2 rendering it incapable of binding CD11b, have normal clotting, but elimination of the CD11b/CD18 binding motif on fibrin(ogen) severely compromises in vivo inflammatory responses triggered by fibrin (92, 96, 98, 109, 110, 111).

## Macrophages and microglia respond directly to fibrin deposits

Binding of fibrin to the CD11b/CD18 and CD11c/CD18 receptors on microglia and brain-infiltrating macrophages activates multiple signal transduction pathways to promote inflammatory responses in the brain (63, 98). The activation results in increased cell body size, rearrangements of the actin cytoskeleton, induction of antigen presentation, release of reactive oxygen species (ROS) and secretion of leukocyte-recruiting chemokines (63, 92, 98, 111).

Fibrinogen deposition in the CNS after BBB disruption induces encephalitogenic immune responses and peripheral macrophage recruitment into the CNS, leading to demyelination. Fibrinogen stimulates a unique transcriptional signature in CD11b+ antigen-presenting cells, inducing the recruitment and local CNS activation of myelin antigen-specific Th1 cells (93, 98, 111). Injection of fibrinogen in the corpus callosum induces microglial activation and demyelination in a model system termed fibrin-induced encephalomyelitis (FIE) (93). Similarly, cortical fibrinogen injection induces CD11b/CD18-microglia-mediated dendrite loss and dendritic spine elimination (32, 112). As described above, transgenic knock-in 390–396A mice express a fully coagulable fibrinogen that selectively lacks the CD11b receptor binding site (92, 109). Injection of plasma derived from these mice does not induce dendritic spine loss, encephalomyelitis or expression of neurotoxic microglia genes, indicating the interaction of P2 with CD11b and CD11c and subsequent microglia activation is responsible for dendrite loss and spine elimination (32, 93, 98, 111).

## Microglia and macrophages mediate neuroinflammation driving AD disease pathophysiology

Single cell RNA-Seq has identified three distinct microglia groups: homeostatic microglia, intermediate disease-associated microglia (DAM1), and disease-associated microglia (DAM2). Microglia transition to activated DAM states in response to amyloid plaques (104). DAM have also been identified by others as microglial neurodegenerative phenotype (113), activation response microglia (114), interferon response microglia (IRM), major histocompatibility complex class II expressing microglia (MHCII+), and cell cycling/proliferating microglia (CPM) (114, 115). While all three sets of microglia express CD11b+, CD11c is only expressed on DAM1 and DAM2 (104). Further, in response to the accrual of Aβ plaques, adjacent microglia increase their expression of CD11b, CD45 and CD68 (116).

Recent human AD GWAS have identified several low penetrance alleles in genes coding for the expression of receptors on the surface of microglia (117, 118), implicating these cells as key mediators of AD disease progression. For example, AD-associated variants have been identified in TREM2 (triggering receptor expressed in myeloid cells 2), which is now recognized as important in microglial and AD biology (119–122). TREM2 is a microglial receptor for Aβ and transduces Aβ -induced downstream signaling (122). DAM are activated sequentially by TREM2-independent and -dependent pathways. In AD mice that lack Trem2, microglia predominantly display the DAM1 state, while DAM2 microglia are virtually absent, suggesting that TREM2 is necessary for the transition to DAM2 (104). Multiple companies are evaluating TREM2 agonists for treating dementia (123). The Anti-TREM2 MAb AL002, produced by the biotech company Alector, is the furthest along, with the Phase 2 INVOKE2 trial for patients with early AD in progress. However, the company reported ARIA in APOε4 homozygous patients treated with AL002, and amended the protocol to exclude them (124).

Additional approaches are being developed to address neuroinflammation in AD (Table 2) (13). These fall into two categories: broadly immunosuppressive agents (e.g., Jak1/2i, p38i, TNFRi, IL-1βi), which have a significant risk of infection and secondary malignancies, and microglia-directed agents (e.g., CSF-1Ri, TREM2 agonist, Semaphorin 4Di, Siglec3i). This latter approach should also be taken with caution, however, as inhibition of microglia phagocytosis results in increased Aβ deposition in the brain of AD mice (125, 126). In contrast to these approaches, targeting fibrin has the potential to selectively eliminate innate immune cell function that induces neurodegenerative and oxidative stress gene signatures, without affecting their homeostatic functions needed to block progression of neurodegeneration (97, 98).

## Genetic and pharmacological studies demonstrate a causal role for fibrin and/or fibrinogen in the development of neurodegenerative diseases

Pharmacologic or genetic depletion of fibrinogen reduces cognitive decline and neuropathology in multiple AD mouse models (18, 24, 65, 71, 99, 127) (Table 3). The 5XFAD mouse is an aggressive genetic model of cerebral amyloidosis routinely used to evaluate different mechanisms of neurodegeneration in AD. 5XFAD mice crossed to 390–396A mice have significantly reduced neuroinflammation, synaptic deficits, and cognitive decline compared to 5XFAD mice (33, 98). Similarly, AD transgenic mice (TgCRND8) which are made heterozygous for the functional fibrinogen αchain (fbγ +/−) are protected from cognitive deficits and BBB damage (24, 67), and fibrinogen deficiency also protects against brain pathology in pericyte-deficient mice (128). Conversely, AD mice made heterozygous for a functional plasminogen gene, which have reduced fibrinolysis, show increased neurovascular damage compared to AD mice (67). These data strongly support the role of fibrin as a potent activator of microglial cells resulting in spine elimination, expression of neurodegenerative and oxidative stress genes and dendritic loss in mice (32, 98).

Recent transcriptome and phosphoproteome profiling of 5XFAD mice indicates upregulation of neurotoxic microglia genes and signaling cascades involved in neurodegeneration and oxidative stress, including phosphorylation of MEK2 and the nicotinamide adenine dinucleotide phosphate (NADPH) oxidase subunit neutrophil cytosolic factor 2 (NCF2). This signature was largely reduced in 5XFAD mice crossed to Fgg 390–396A mice, indicating that it is induced by fibrin P2 (97, 98).

Changes in clotting and the fibrinolytic system contribute to vascular dysfunction, inflammation, coagulation, and cognitive impairment in AD (129). Pharmacological interventions that affect fibrin directly or indirectly can affect neurodegeneration in mouse models of AD. For example, depletion of fibrinogen with the snake venom enzyme ancrod lessens BBB dysfunction and protects or resolves neurological pathology in animal models of AD (67, 130). The fibrinogen-derived γ377−395 P2 peptide competitively inhibits fibrin’s interaction with CD11b, and presumably CD11c, in both in vitro biochemical experiments (131) and microglial activation assays (92). Intranasal administration of the fibrin P2 γ377–395 peptide reduces brain parenchyma Aβ deposition and protects from cognitive deficits in AβPP/PS1 AD mouse model (132). Moreover, administration of the γ377–395 peptide does not affect blood coagulation (92, 132).

tPA is a serine protease that converts plasminogen into plasmin, an enzyme involved in fibrin degradation. tPA interacts with Aβ and localizes with plasminogen in Aβ plaques in AD mice (AβPPP, Tg2576) (133, 134). Levels and activity are reduced in two AβPPP mouse lines (TgCRND8 and Tg2576) (135). Indeed, tPA-deficient mice show delayed clearance of injected Aβ, associated with microglial cell activation and neuronal damage (135). Conversely, recombinant tPA attenuates AD-related pathology in AD transgenic mice by reducing cerebral Aβ levels and improving the cognitive function (136). A recent GWAS study identified α−2-antiplasmin (SERPINF2), an inhibitor of plasmin, as a new genetic locus linked with AD (137).

Direct oral anticoagulants (DOACs) targeting thrombin have been proposed as therapeutics for AD (138, 139). Blocking the conversion of fibrinogen into fibrin with anticoagulants is protective against inflammation and cognitive impairment in AD mice (140–143). Long-term treatment of AD mice with the anticoagulant dabigatran prevents cerebral fibrin deposition and cognitive decline, and significantly reduced the extent of amyloid plaques, oligomers, phagocytic microglia, and infiltrated T cells (144). Accordingly, long-term treatment of AD mice with RU-505, which blocks fibrin-Aβ clots, reduced vascular amyloid deposition, vessel infarctions, cerebral microgliosis, as well as cognitive impairment (127, 145). In a retrospective study on ~440,000 patients, oral anticoagulants decreased the risk of many types of dementia by 48% in patients with arterial fibrillation (146).

## An anti-fibrin P2 MAb is effective in animal models of AD

To further evaluate fibrin as a therapeutic target for neurological diseases, Ryu et al. developed mouse MAb 5B8 (99), which selectively target the cryptic fibrin P2 epitope required for CD11b binding without adverse effects in coagulation (Figure 2). In animal models of both MS and AD, 5B8 entered the CNS and bound to parenchymal fibrin. The therapeutic utility of 5B8 was evaluated in three models of EAE, which simulate key aspects of MS. Prophylactic or therapeutic administration of 5B8 protected mice from axonal damage and reduced disease severity compared to isotype-matched controls. This fibrin-targeting immunotherapy also protected in genetic rodent AD models. In 5XFAD mice, 5B8 co-localized with fibrin-rich areas surrounding Aβ plaques, demonstrating 5B8 penetration of the BBB. Treatment with 5B8 reduced the loss of cholinergic neurons and microglial activation around plaques and reduced the expression of inflammatory and oxidative stress genes. 5B8 blocked activation of the nicotinamide adenine dinucleotide phosphate (NADPH) oxidase and reduced ROS in microglia and macrophages (99). In initial phosphoproteomic profiling studies, 5B8 blocked fibrin-induced MEK2 phosphorylation in bone marrow-derived macrophages (98). Co-expression analysis revealed in the brain of 5XFAD 5B8-downregulated genes encoding molecules of the complement pathway, including C4b and C1q, as well as Trem2 and Tyrobp, which encodes a co-receptor for CD11b (99). Indeed, Tyrobp was one of the genes with the greatest degree of downregulation by 5B8 in 5XFAD brain (99). Overlay with networks of genes encoding noninflammatory molecules from brains of humans with AD showed that 5B8 downregulated 65% of the Tyrobp-related network. Overall, these results support that 5B8 suppresses innate immune pathogenic pathways that mediate amyloid-related neurodegeneration.

Importantly, these studies also showed that targeting P2 was safe, that 5B8 did not alter the polymerization of fibrinogen into fibrin, did not inhibit the activated partial thromboplastin time (aPTT) in human plasma, and had no effect on the clotting time of mouse plasma in vivo (99). Mice deficient in P2, as well as mice treated with 5B8, are not immunocompromised and can be housed in conventional animal facilities without opportunistic infection (99, 109). Similarly, in humans, congenital afibrinogenemia, a genetic disorder characterized by a complete absence of fibrinogen, is associated with excessive bleeding but no increase in opportunistic infections (147). The selectivity and efficacy of 5B8 in animal models strongly support the development of a MAb drug targeting fibrin-P2 peptide for the treatment of neuroinflammatory diseases.

## THN391, a first-in-class anti-fibrin antibody developed for clinical testing for the treatment of dementia and other inflammatory neurodegenerative diseases

**Figure 3.**
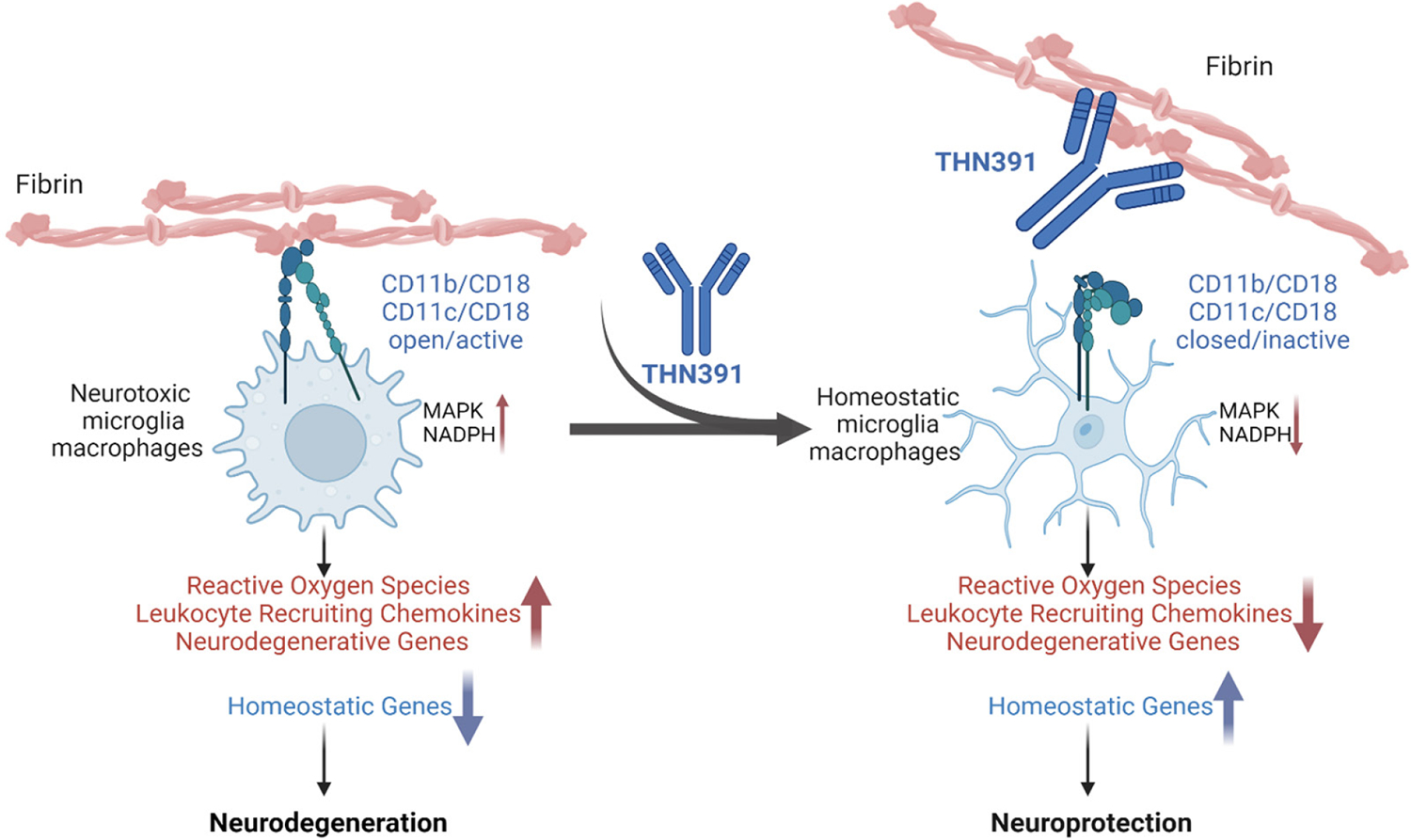
Fibrin-targeting immunotherapy selectively blocks the neurotoxic inflammatory response Fibrin binding of the exposed fibrin P2 sequence to the α-I domain of CD11b and CD11c on microglia, monocytes, macrophages, and dendritic cells triggers an inflammatory neurotoxic response, including induction of oxidative stress, leukocyte recruitment and neurodegenerative pathways (32, 92, 97–99, 111) Working model of fibrin-targeting immunotherapy THN391 binding to fibrin P2 and blocking this response. Created with BioRender.com.

Therini Bio developed the clinical candidate THN391, a humanized affinity-matured MAb derived from murine MAb clone 5B8 (Figure 3). It was tested across a similar set of in vitro and in vivo studies used to characterize 5B8. Affinity maturation resulted in an ~100-fold greater binding affinity for human P2 (sub-nanomolar). THN391 does not bind to soluble fibrinogen. THN391 can block binding of the CD11b and CD11c αI domain to fibrin P2. The Fc region was engineered to remove FcγR binding, rendering THN391 a purely antagonistic agent incapable of mediating antibody effector functions, i.e., antibody-dependent direct cell killing and antibody-dependent cell phagocytosis. Importantly, THN391 does not alter the normal coagulation process as measured by aPTT and thromboelastography in ex vivo experiments (Kantor et al., manuscript in preparation).

THN391 was safe and well-tolerated in preclinical and nonclinical safety studies (148). There were no effects of THN391 on coagulation, no adverse effects observed on the CNS in either rats or monkeys, and no THN391-related adverse effects were observed on the cardiovascular and respiratory systems in monkeys with IV doses up to 100 mg/kg. Furthermore, there were no signs of global immunosuppression or increased infection following two months of dosing in 4-week rat and NHP GLP toxicology studies (up to 100 mg/kg). Plasma PK data from mouse, rat, and monkey were used to predict human plasma PK following a single dose and multiple doses. Consistent with the typical PK profile of MAb therapeutics, the model predicts a long plasma, CSF, and ISF terminal elimination phase following single and every 4-week (Q4W) dosing.

The antibody treatment prevents further inflammation and therefore limits the vascular damage due to ROS and deposition of fibrin (Kantor et al., in preparation). By inhibiting the feed-forward mechanism, THN391 may have the added benefit of reducing the amount of fibrin in the brain. Furthermore, anti-Aβ therapy can promote phagocytosis and removal of Aβ plaques embedded in the microvasculature, which likely underlies the risk of ARIA associated with this approach. In contrast, by blocking rather than removing these deposits, it is predicted that THN391 will carry less risk of microhemorrhage. By targeting fibrin with a purely antagonistic antibody, THN391 has the potential to target both Aβ-associated and non-associated fibrin.

Therini Bio recently initiated a first-in-human phase 1 study to assess THN391 safety and tolerability following intravenous administration «A Double-blind, Randomized, Placebo-controlled Phase 1 Study to Assess the Safety Tolerability, and Pharmacokinetics of Single and Multiple Ascending Doses of THN391 in Healthy Subjects» (15, 148). The primary goal of this study is to define THN391 initial safety and tolerability profile following intravenous administration. Emphasis on potential hemostatic effects is being sought through the inclusion of special safety studies, including thromboelastography. To test the efficacy of THN391 in AD, Phase 2 studies will be required for collection of safety endpoints, pharmacodynamic and exploratory clinical endpoints in AD patients.

## Advantage of targeting fibrin over other anti-inflammatory approaches

Fibrin P2, a vascular-derived upstream driver of pathogenic innate immune activation, is not currently targeted by any other therapy. Similar to its mouse precursor 5B8 (32, 99), THN391 inhibits the generation of neurotoxic microglia and the infiltration of pathogenic macrophages in the brain (Kantor et al., manuscript in preparation). Other anti-inflammatory approaches do not inhibit the neurotoxic reprogramming of innate immune cells or peripheral macrophages or neutrophils that are critical mediators in autoimmune diseases and traumatic injuries in brain and periphery. Unlike global inhibitors of innate immune cells, THN391 should not interfere with protective functions of microglia or clearance of Aβ. This unique combination of selectivity with potency is a potential advantage of targeting fibrin compared to other strategies to interfere with microglia or macrophage functions.

THN391 is designed to have clinical benefits in a broad set of patient populations, including those with neurodegenerative dementia, autoimmune diseases (MS), diabetic retinopathy, age-related macular degeneration, and traumatic injuries. Multiple preclinical studies in animal models of diverse etiologies that are protected or rescued by eliminating fibrin-induced activation support this concept (Table 3 (92, 99, 149) and Kantor et al, in preparation). Given the high overlap between sporadic CAA and AD, THN391 could be considered for treatment of both. THN391 may also be applicable to subjects with neurodegeneration and dementia in the absence of Aβ (suspected non-Alzheimer’s pathophysiology (SNAP)) (150). Activation of microglia and neuroinflammation contribute to pathology in dementia with Lewy Bodies (151) and frontotemporal dementia (152, 153), indicating these conditions may also be responsive to THN391.

## Potential for combination treatments

The complex pathophysiology of AD may require combination treatments rather than monotherapy. Fibrin-induced activation of the innate immune response is an independent and orthogonal pathway of neurotoxicity that is currently not targeted directly by any other therapeutic approach. The anti-Aβ MAbs currently used for the treatment of AD all lower Aβ burden but are only somewhat effective in slowing cognitive decline (1–3). Given the incidence of ARIA, the risk-benefit profile of these drugs is only moderately favorable. Treatment of AD with THN391 alone or in combination with anti-Aβ antibodies, perhaps at lower doses, has the potential to be more effective in slowing cognitive decline and reducing the incidence of ARIA. Anti-TREM2 MAb therapies target only disease-associated microglia and similarly carry a risk of ARIA in APOE4 homozygotes. In contrast, THN391 blocks the interaction of fibrin with a broader set of microglia, macrophages and dendritic cells, and may be more effective.

THN391 targets the toxic effects of fibrin. Given the incidence of ARIA due to microhemorrhage, it is conceivable that the risk can be reduced by counter acting the resulting fibrin deposition due to the microhemorrhage. Either coadministration or sequential dosing of THN391 could significantly reduce the concomitant neuroinflammation that can occur with ARIA.

While the approval of the anti-amyloid lecanemab for patients with MCI and mild Alzheimer’s disease is encouraging, much effort is still needed to develop treatments that have a significant impact on cognitive decline. THN391 provides a potential novel approach by targeting fibrin as a driver of neurodegeneration in dementia. It should be effective in inhibiting neuroinflammation, reducing amyloid-driven neurotoxicity and hopefully slowing the decline of cognitive ability in dementia patients. Either alone or in combination with anti-amyloid therapy, it may provide a new opportunity to enhance the therapeutic toolbox for physicians treating patients with dementia.

## Figures and Tables

**Table 1.** Fibrin(ogen) in Alzheimer’s and related diseases

Subject	Evidence	References
A. Neuropathology		
Human	COX2+ macrophages infiltrate the perivascular spaces of the AD brain and correlate with perivascular fibrinogen leakages, interruptions of the endothelial tight junction protein ZO1 and Aβ plaques.	(154)
Human	Insoluble fibrinogen–albumin complexes exist in AD brains.	(155)
Human Rat	Fibrin in the perivascular area associates with Aβ plaques, microglia, microglial activation and astrogliosis.	(28)
Human TgCRND8, 5XFAD	Fibrin(ogen) is deposited parenchymally in CAA-positive vessels and associates with Aβ.	(24, 32, 33)
AppNL-G-, F/NL-G-F	An anti-insoluble fibrin antibody that does not bind to fibrinogen or fibrin degradation products identified deposits around and inside blood vessel walls and colocalization with Aβ in the small capillaries and arterioles. The amount of both proteins in lesions positively correlated with disease stage and severity.	(77)
Human	Fibrin(ogen) deposition was observed in AD cases and there was a strong correlation with CAA severity. Deposition was highest in AD patients and cases homozygous for APOEe4.	(27)
Human	Perivascular fibrin deposits correlated with loss of pericyte coverage of brain vessels.	(29)
Human TgCRND8	Fibrin deposition increases in the AD brain and correlates with the degree of pathology.	(62)
Human	Fibrin accumulation in the parietal cortex is correlated with PDGFRβ loss and fibrillary accumulation.	(31)
Human	CAA-linked β-amyloid mutations promote cerebral fibrin deposits via increased binding affinity for fibrinogen.	(66)
Tg6799	Striatal fibrinogen extravasation and vascular degeneration correlate with motor dysfunction in an aging mouse model of AD.	(156)
Human	APOE4 increases pericyte degeneration, BBB breakdown, cerebral amyloid angiopathy, and fibrinogen deposits in the brain.	(46)(26)
B. Biomarkers		
Human	High plasma fibrinogen is associated with an increased risk of AD and vascular dementia.	(79, 80)
Human	Fibrinogen in the CSF and plasma can serve as a useful biomarker to identify AD progression.	(83–85) (86, 87)
Human	CSF fibrinogen levels are reported to correlate with other CSF biomarkers of BBB breakdown including QAlb and sPDGFRβ.	(43, 46, 59, 60)

**Table 2.** Selected anti-inflammatory compounds currently in clinical trials for AD

Compound	Target and Mechanism of Action	Type	Stage
Masitinib	Tyrosine kinase inhibitor exhibits neuroprotection via inhibition of mast cell and microglia/macrophage activity	SM	Phase 3
NE3107	Beta-androstenetriol with anti-inflammatory and insulin signaling effects via ERK 1 and 2	SM	Phase 3
AL002	Targets TREM2 on a subset of microglial cells	MAb	Phase 2
BCG Vaccination	Bacillus Calmette–Guérin vaccination for immunomodulation	Live Attenuated Mycobacterium	Phase 2
Baricitinib	Janus kinase (JAK) 1/2 inhibitor	SM	Phase 2
Canakinumab	IL-1β inhibitor	MAb	Phase 2
CY6463	Guanylate cyclase positive allosteric modulator, synaptic plasticity/neuroprotection	SM	Phase 2
Daratumumab	CD38 inhibitor/depletion, immunomodulatory effects	MAb	Phase 2
Lenalidomide	Inhibition of TNF-α and other inflammatory cytokines	SM	Phase 2
Montelukast	Leukotriene receptor antagonist (LTRA); anti-inflammatory effects	SM	Phase 2
MW50	Kinase inhibitor selective for p38 MAPKα	SM	Phase 2
Neflamapimod VX-745	Kinase inhibitor selective for p38 MAPKα; affects microglial activation	SM	Phase 2
Pepinemab	Semaphorin 4D/CD100 inhibitor, reduces inflammatory cytokine release	MAb	Phase 2
TB006	Inhibits galectin 3, a β-galactosidase-binding protein that activates macrophages and inflammation	MAb	Phase 2
Tdap	Tetanus toxoid, reduced diphtheria toxoid, and acellular pertussis vaccine to stimulate inflammatory protection	Bacterial components	Phase 2
Sargramostim	Hematopoietic growth factor GM-CSF; anti-inflammatory. activate microglia	rHu	Phase 2
XPro1595	Second- generation selective TNF inhibitor that neutralizes soluble TNF without affecting trans-membrane TNF	TNF analog (mutein)	Phase 2
AL003	Inhibits CD33/Siglec 3, an inhibitory microglial transmembrane receptor that interacts with TREM2	MAb	Phase 1
CpG101	Toll-like receptor 9 agonist leading to reduced Aβ plaques and tau pathology	NA Oligo	Phase 1
Edicotinib JNJ-40346527	CSF-1R inhibitor, microglial modification	SM	Phase 1
Emtricitabine	Nucleoside reverse transcriptase inhibitor (NRTI); reduces neuroinflammation by target inflammasome upstream IFN signaling	SM	Phase 1
Salsa late	Non-steroidal anti-inflammatory (NSAID); inhibit p300 acetyltransferase and by that reduce tau acetylation	SM	Phase 1
VT301	Autologous AD specific regulatory T cells (CD4+ and CD25+ Tregs)	Cell Therapy	Phase 1

Selectively adapted from recent reviews (13) and (14). SM = small molecule; MAb = monoclonal antibody, NA = nucleic acid; rHu = recombinant human; TNF = tumor necrosis factor, MOA = mechanism of action.

**Table 3.** Genetic and pharmacological evidence supporting anti-fibrin therapy in dementia

Strain	Evidence	References
A. Genetic		
TgCRND8 PDAP Tg2576 Fgα+/−	AD transgenic mice heterozygous for fibrin Fga gene have reduced BBB damage and reduced microglial activation	(67)
	AD mice heterozygous for the plasminogen gene have reduced fibrinolysis and increased neurovascular damage	
TgCRND8 Fgα+/−	AD transgenic mice heterozygous for Fga have reduced amyloid pathology and are protected from cognitive deficits	(24)
TgCRND8 Fga+/−	AD transgenic mice heterozygous for fibrin Fga gene a decreased in fibrinogen levels and reduced amyloid pathology	(62)
Fga+/− PdgfrbF7/F7	Fga heterozy city reduces pathology in pericyte-deficient mice	(128)
5XFAD Fgg390–396A	5XFAD mice crossed to Fgg390–396A mice, which no longer have fibrin P2, have significantly reduced neuroinflammation, synaptic deficits, and cognitive decline compared to 5XFAD mice	(32)
5XFAD Fgg390–396A	Multiomic profiling reveals fibrin neurodegeneration and oxidative stress signature in 5XFAD mice but not controls. This signature is reduced in (5XFAD X Fgg390–396A) mice.	(98)
B. Pharmacological		
Tg6799 TgCRND8	RU-505 inhibits Aβ-fibrinogen interaction, reduces vascular amyloid deposition and microgliosis and improves cognitive impairment in AD mice	(145)
TgCRND8	Fibrinogen depletion with ancrod diminishes neuroinflammation and vascular pathology.	(67)
	Treatment with the plasmin inhibitor tranexamic acid, which blocks fibrinolysis, aggravates the pathology. Pretreatment with ancrod reduces the increased pathology from plasmin inhibition.	
PdgfrbF7/F7	Fibrinogen depletion with ancrod ameliorates pathology in pericyte-deficient mice	(128)
AβPP/PS1	The fibrinogen-derived γ377−395 P2 peptide competitively inhibits the interaction of CD11b, and Intranasal administration protects from cognitive deficits in AD mice	(132)
APPswe/PS1	tPA converts plasmin to plasminogen, promoting fibrinolysis. Recombinant tPA attenuates AD-related pathology by reducing cerebral Aβ levels and improving the cognitive function.	(136)
	Conversely, increasing plasmin activity with α−2-antiplasmin antisense oligonucleotide treatment exacerbates the brain’s immune response and plaque deposition.	
TgCRND8	Long-term treatment with the thrombin inhibitor dabigatran prevents cerebral fibrin deposition and cognitive decline. It significantly reduces the extent of amyloid plaques, oligomers, phagocytic microglia, and infiltrating T cells.	(144)
Thy1-YFP	Fibrinogen and plasma injection into the cortex induce dendrite loss and spine elimination in the healthy brain.	(32)
5XFAD	Anti-fibrin P2 MAb 5B8 is effective in a mouse model of AD	(99)
5XFAD	MAb 5B8 blocks p-MEK2 in fibrin-treated bone marrow-derived macrophages	(98)

APP = amyloid precursor protein; PS1 = presenilin 1; tPA = tissue-plasminogen activator
